# Third Cranial Nerve Palsy Due to COVID-19 Infection

**DOI:** 10.7759/cureus.14280

**Published:** 2021-04-03

**Authors:** Steven Douedi, Hani Naser, Usman Mazahir, Amin I Hamad, Mary Sedarous

**Affiliations:** 1 Internal Medicine, Jersey Shore University Medical Center, Neptune, USA; 2 Pulmonary and Critical Care Medicine, Jersey Shore University Medical Center, Neptune, USA; 3 Internal Medicine, October 6 University, Cairo, EGY; 4 Neurology, Jersey Shore University Medical Center, Neptune, USA

**Keywords:** coronavirus, covid-19, cranial nerve, nerve palsy, ptosis, oculomotor nerve (cn iii) palsy

## Abstract

Coronavirus disease 2019 (COVID-19) is known to be primarily a viral infection affecting the pulmonary system leading to severe pneumonia and acute respiratory distress syndrome. COVID-19 has also been found to affect the neurological system causing various nerve palsies. While some studies have suggested these neurological manifestations may indicate severe disease, cranial nerve palsies in the setting of COVID-19 infection have been linked to improved patient outcomes and mild viral symptoms. We present a case of a 55-year-old male with confirmed COVID-19 infection presenting with third cranial nerve palsy. Since his hospital course remained unremarkable, he was treated supportively for his COVID-19 infection and remained stable on room air during his hospitalization. No causative factors other than COVID-19 were identified as a cause for his cranial three nerve palsy which resolved spontaneously during outpatient follow-up. Although different cranial nerve palsies associated with COVID-19 infection have been identified in the literature, the pathogenesis and prognosis of cranial nerve palsy is still unclear. This case emphasizes the need for continued symptom monitoring and identification in patients diagnosed with COVID-19.

## Introduction

The third cranial nerve, or oculomotor nerve, supplies several extraocular muscles as well as the ciliary muscle, sphincter pupillae, and levator palpebrae superioris [1]. While there are several causes of third cranial nerve palsy, the most common have been identified as hypertension and diabetes mellitus [1,2]. Coronavirus disease 2019 (COVID-19) is known to be primarily a viral infection affecting the pulmonary system leading to pneumonia, acute respiratory distress syndrome, and even subcutaneous emphysema in severe cases [3,4]. Although uncommon, COVID-19 has also been found to affect the neurological system causing various nerve palsies [5-7]. We present a case of a male with confirmed COVID-19 infection presenting with third cranial nerve palsy.

## Case presentation

**Figure 1:**
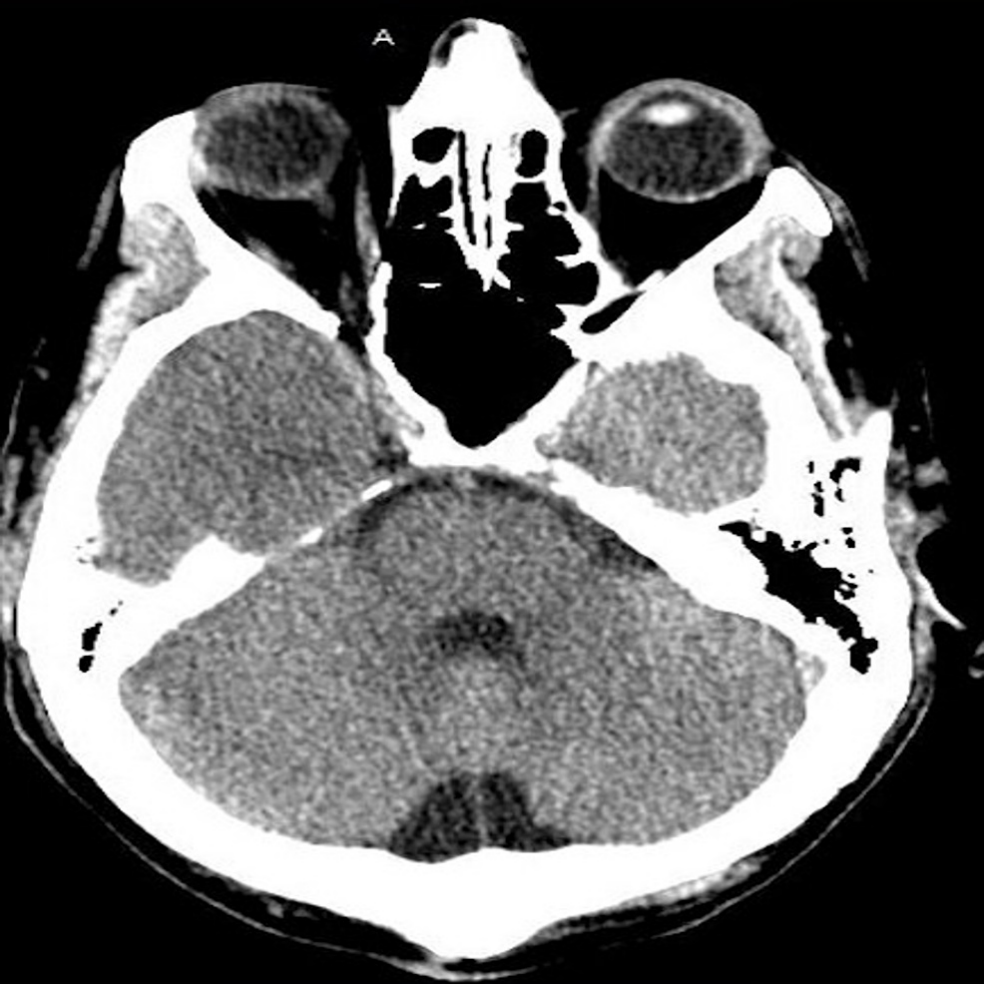
CT scan of the head without contrast. Unremarkable CT scan of the head showing no intracranial pathology.

A 55-year-old male with only a remote medical history of seizure disorder on levetiracetam presented to the emergency department (ED) complaining of a generalized and bilateral headache graded a 2-3/10 for the past six days prior to admission. He had generalized fatigue, a loss of sense of taste, and double, blurry vision since this time period. He had exposure to a family member who was COVID-19 positive prior to symptom onset. He denied any other medical history including hypertension or diabetes mellitus (hemoglobin A1c on admission was 5.8%) and denied any significant family or social history. In the ED, he was evaluated by the neurologist and ophthalmologist. Physical exam revealed a blood pressure of 122/79 mm Hg, heart rate of 89 beats per minute, respiratory rate of 16 breaths per minute, oxygen saturation of 98% on room air, and temperature of 98 degrees Fahrenheit. His pupils were reactive to light bilaterally but noted to have left sided ptosis and diplopia on all fields of gaze except to the left. His visual acuity was 20/50 in both right and left eyes. He was noted to have 10% reduction of adduction and elevation in his left eye. Facial nerve sensation was intact in all branches and facial strength was symmetric. There was no evidence of pupil or orbital involvement on slit lamp or fundal ophthalmologic examination. He was also noted to have mild bilateral lower extremity sensory deficits with intact proprioception which he claimed to be chronic due to an old injury several years prior to this admission. He had no other sensory or neurological deficits on physical examination. He was determined to be COVID-19 positive using reverse transcription-polymerase chain reaction (RT-PCR). Complete blood count and comprehensive metabolic panels were all within normal limits. A computed tomography (CT) scan of the head without contrast was emergently obtained but was unremarkable (Figure 1). 

**Figure 2:**
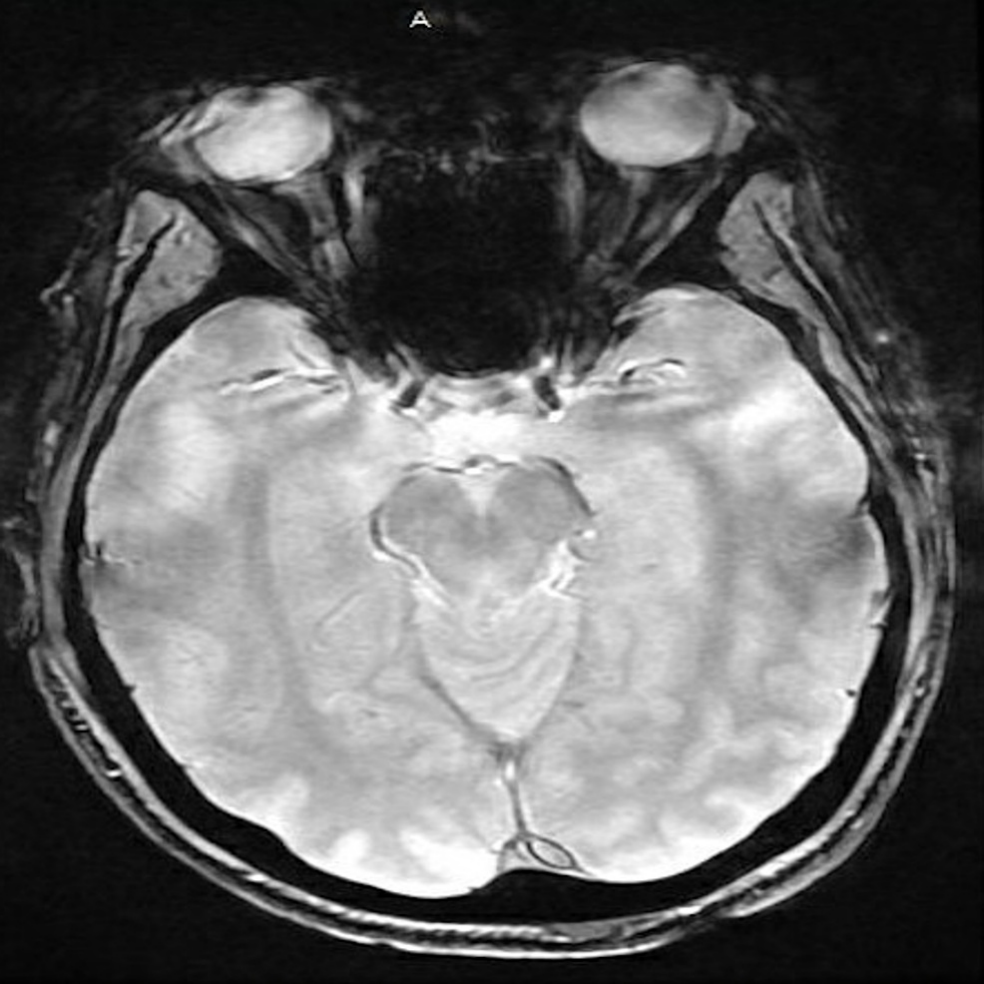
MRI of the brain. Unremarkable MRI scan of the brain showing no intracranial pathology.

Further testing with CT angiography and magnetic resonance imaging (MRI) of the brain and neck were also unremarkable without any signs of nerve compression, masses, or aneurysms (Figure 2).

He was admitted for further observation and was treated supportively for COVID-19 and third cranial nerve palsy. On day 3 of hospitalization, his symptoms gradually improved and he was discharged with outpatient neurologic and ophthalmologic follow-ups. One week after discharge his symptoms continued to improve and ultimately his ptosis and diplopia completely resolved.

## Discussion

COVID-19 has been one of the largest and detrimental pandemics with significant morbidity and mortality [3]. Primarily identified as a respiratory illness, COVID-19 has been known to cause a wide range of symptoms including neurological features. It has been suspected that COVID-19 penetrates the cribriform lamina of the ethmoid bone, leading to anosmia and acts as an entry point to the nervous system [8,9]. The virus’s affinity to the angiotensin-converting enzyme-2 (ACE-2) receptor, which is found in the oral and nasal mucosa as well as the nervous system, may likely be the cause of neurological involvement in these patients; yet the exact pathogenesis still remains unknown [5,9]. 

Third cranial nerve palsy has been associated with various comorbidities, most commonly hypertension and diabetes mellitus, and has also been found in the setting of trauma or nerve compression [1]. Our patient, despite having a remote history of seizure disorder, did not have any known contributing factors or possible causes for his third cranial nerve palsy other than being infected with COVID-19. Our patient's MRI and CT brain scans were unremarkable for tumors, aneurysms, or intracranial bleeds further increasing suspicion for COVID-19 induced pathology. Although Mao et al. reported up to 36% of COVID-19 infected patients experience neurological symptoms which were associated with more severe disease [10]. In our patient and several others with cranial nerve palsy, COVID-19 symptoms were mild and neurological changes resolved over time without specific intervention [5,7,8]. This may suggest that cranial nerve palsy in the setting of COVID-19 infection may be associated with improved patient outcomes and temporary cranial nerve deficits compared to other neurological deficits. Further and larger studies are needed to establish this association and define the prognosis of COVID-19 infection and neurological symptoms. 

## Conclusions

Although different cranial nerve palsies associated with COVID-19 infection have been identified in the literature, the pathogenesis and prognosis of cranial nerve palsy is still unclear. It is also questionable if third cranial nerve palsy may be a good prognostic indicator as it is associated with mild COVID-19 disease in our patient and others in the literature. As COVID-19 continues to advance and spread, its wide range of symptoms and manifestations are still being discovered. This case emphasizes the need for continued symptom monitoring and identification in patients diagnosed with COVID-19.
